# Affective work rumination as a mediator of the reciprocal relationships between job demands and exhaustion

**DOI:** 10.1371/journal.pone.0293837

**Published:** 2023-11-09

**Authors:** Martin Geisler, Sandra Buratti, Carl Martin Allwood

**Affiliations:** Department of Psychology, University of Gothenburg, Gothenburg, Sweden; Universidad Central de Chile, CHILE

## Abstract

High levels of job demands are considered as the main predictor for teachers’ exhaustion, but longitudinal studies of the causal effects are few. Recently it has been suggested that research should further explore possible reciprocal relationships between stressors and strain and investigate if work rumination contributes to explain these relationships. In a sample of teachers (n = 1067) using a three-wave design, we hypothesized positive causal effects of job demands (work pace and role conflict) on affective work rumination, and of affective work rumination on exhaustion. We also hypothesized a positive reversed causal effect of exhaustion on affective work rumination, and of affective work rumination on job demands. Furthermore, affective work rumination was expected to mediate the positive causal and reversed causal effects between job demands and exhaustion. The results partly confirmed the expected causal and reversed causal effects. However, affective work rumination was only found to mediate the reversed causal effect of exhaustion and role conflict. Furthermore, a reciprocal relationship was only found between role conflict and exhaustion. The empirical, theoretical, and practical implications of the study are discussed.

## Introduction

The teaching profession is a high-stress occupation [1–4]. High levels of job demands are persistently reported as the main determinant for teachers’ exhaustion [5–7]. Overall, role conflict and work pace are two specific job demands that seem to be especially important for understanding teachers’ exhaustion [2, 8]. Illustrative of this, both research and labor unions report that teachers express that their professional role has become too diverse and demanding, leading to increased experiences of being confronted with conflicting demands and not having enough time to do the work [5, 9]. In Sweden, the challenging conditions for teachers are apparent with frequent reports of a problematic situation in terms of stress-related ill health during the last decade [9–11]. In fact, the problems seem to be accentuating, with high levels of stress-related absenteeism, prevalence of exhaustion and turnover [12]. In addition, there is a lack of teachers in the Swedish school system and recent projections indicate that this problem will increase in the coming years [13]. High work pace (due to high workload) and role conflict (due to lack of possibility to get relief and assistance for requirements that lie outside the teacher assignment), are considered as key factors for understanding stress-related ill health, turnover, and the lack of staffing of teachers [14, 15].

Research based on Job Demands-Resources theory (JD-R) has contributed to the understanding of occupational stress, exhaustion, and burnout in general [16, 17], and among teachers specifically [18]. The basic proposition of JD-R theory is that all work characteristics can be categorized as job demands or job resources [19]. Job demands are those characteristics of the job that require effort, are obstructive, and trigger the health-impairment process—whereas job resources pertain to characteristics of the job that contribute to personal growth and achievement, thereby eliciting the motivational process. Specifically, it is postulated that job demands can initiate a health-impairment process when workers’ daily exposure to high levels of demands, over time, leads to chronic overload, and chronic exhaustion [20]. Thus, JD-R theory propose that job demands are the most important predictors for negative outcomes of the health-impairment process, such as exhaustion [17]. Recent developments in JD-R theory propose that, depending on their occupational health and well-being, employees may influence their work environment in either a constructive or a deteriorative way [20]. Regarding the health-impairment process, employees who face higher levels of demands become stressed, experience strain, and are at risk of engaging in various types of undermining behavior, which instigate a reciprocal loss cycle in terms of increased levels of stress, ill health, and demands at work [21, 22]. Furthermore, more recent expansions specifically integrate a self-regulation perspective into JD-R theory—proposing that the more job strain *or* burnout symptoms individuals experience the more likely they are to use maladaptive coping strategies. This maladaptive behavior is suggested to be caused by relapsing burnout symptoms such as feelings of exhaustion, impaired cognitive functioning, and negative mood [23].

The proposition that job demands leads to exhaustion is at the core of JD-R theory, but the association has mostly been reported in cross-sectional studies [18]. Acknowledging the lack of a detailed examination of the empirical evidence supporting the basic assumptions of JD-R theory over time, Lesener et al. [24] conducted a meta-analytical review of longitudinal studies based on JD-R theory. The results supported the basic assumption in JD-R theory: that job demands (at Time 1) predicted burnout (at Time 2). However, separate analyses of high-quality studies showed that a reciprocal model had the best fit to the data, and that burnout was a clear predictor of job demands, whereas the effect of job demands on burnout was less clear. Furthermore, another recent meta-analysis of longitudinal studies by Guthier, Dormann, and Voelke [25] investigated reciprocal effects between stressors and strain. Corroborating the reports of Lesener et al. [24], Guthier et al. [25] found evidence of reciprocal effects between stressors (job demands) and strain (exhaustion), and that the effect of strain on stressors seems to be more evident than the effect of stressors on strain.

The meta-analytical reviews by Lesener et al. [24] and Guthier et al. [25] demonstrate support for the health-impairment process as described in JD-R theory, and the importance of attending to reciprocal effects for understanding occupational health (cf. [26]). However, the reports also highlight some issues and limitations that need to be addressed. Lesener et al. [24] advanced the need for more research based on a minimum of three-wave data, using time intervals that are theoretically and methodologically appropriate for the specific study, and that address reversed and reciprocal relationships between job characteristics and well-being. Also, Guthier et al. [25] stated that the results of their meta-analysis are not conclusive, as most primary studies only used two measurement occasions and time intervals longer than twelve months. Furthermore, Guthier et al. [25] noted that a possible explanation for the strain effect could be that stress reactions may become chronic when a person is unable to experience recovery and that further elevated stress levels and exhaustion can be caused by job stressors—whether they are present or not (e.g., anticipated and the focus of work ruminative thinking: cf. [27]). Thus, Guthier et al. [25] recommended that future research should investigate extended job stress models that examine the role of recovery experiences to break the reciprocal relationship between stressors and strain. Theoretically, this implies that experiences that enhance and prolong the negative effect of stressors, such as engaging in affective work rumination, may be critical to consider for understanding the reciprocal, health-impairing, relationship between stressors and strain. Specifically, affective work rumination is defined as pervasive, intrusive, and recurrent thoughts directed to negative and dysfunctional emotions related to work and work-related issues–ultimately obstructing recovery by prolonging stress-related physiological activation [28]. That is, affective work rumination can be considered as a manifestation of maladaptive self-regulation to cope with high job strain *or* feelings of exhaustion which, in turn, may lead to accumulating strain and exhaustion (c.f. [23]).

Indeed, some research suggests that the effect of job demands on exhaustion may also be indirect, through the elicitation of tendencies to engage in work rumination during non-work time [1, 29]. For teachers, reports of role conflict and high work pace imply that the professional requirements are perceived to be unclear and difficult to comply with, and that there is not enough time to attend to the disparate demands at work. Over time, these stressful conditions at work may trigger affective work rumination which prolongs and accentuates the effect of these job demands, and ultimately leads to increased feelings of exhaustion (c.f. [25]. In some support of this notion, afternoon anticipation of a high workload the next workday has been reported to be positively associated with day-specific exhaustion before going to work the next morning, when people also reported having engaged in work-related worries during the evening [27]. However, teachers who already experience exhaustion may also be more prone to engage in affective work rumination [30], and, in turn, be more susceptible and inclined to experience higher levels of role conflicts and work pace at work (c.f. [20, 23]).

Based on these considerations, and the recommendations for longitudinal investigations [24, 25], the present study use a three-wave design and investigate the role of affective work rumination for the stressor and strain relationship. Specifically, a three-wave design makes is possible to test both causal and reversed causal effects. The purpose is to investigate causal and reciprocal relationships between job demands (role conflict and work pace), affective work rumination, and exhaustion among teachers. One aim of the study is to contribute to the literature by investigating causal effects of job demands on exhaustion in the specific occupational setting of teachers [18]. Another aim is to contribute to JD-R theory by investigating if affective work rumination mediates the reciprocal relationships between job demands and exhaustion. Specifically, if affective work rumination is a psychological mechanism that is involved in the health impairment process and may contribute to a more fine-grained explanation of the association between job demands and exhaustion [17, 23].

### Job demands and exhaustion–the role of work pace and role conflict for teachers’ exhaustion

As noted, JD-R theory posits that job demands ultimately relate to exhaustion and ill health [17]. However, even if the effect of job demands on exhaustion can be observed on average (e.g., across occupational groups), the effect of specific job demands on exhaustion in certain professions may be unique and therefore important to investigate [31].

Numerous cross-sectional studies report that high levels of job demands are of particular importance for understanding teachers’ exhaustion [3, 5, 6, 32, 33]. Several studies suggest that it is certain tasks and characteristics of the job such as high work pace, administrative and complementary obligations (cf. role conflict) that lead to teachers’ frustration and, ultimately, exhaustion [4, 7, 8, 34, 35]. To confront conflicting demands (i.e., experiencing role conflicts) and not have enough time to do the work (i.e., high work pace) cost effort and drain teachers’ energetic resources, over time initiating a health-impairment process. Some research has investigated the longitudinal effect of job demands on teachers’ exhaustion. In a two-wave study, Lorente et al. [2] investigated the effects of various job demands on teachers’ burnout over time, controlling for baseline levels of burnout. They found that only quantitative demands influenced exhaustion. In a study of beginning teachers, levels of a teacher-specific job demand (classroom disturbance) explained emotional exhaustion two years later [36]. However, in a two-wave study (Time 1-Time 2, separated by 6–9 months), teaching-specific job demands were not found to have a direct effect on exhaustion [37]. Reviewing research on teachers’ well-being based on JD-R theory up to 2014, Taris et al. [18] only found three studies that had used a longitudinal (i.e., two-wave) design. Out of these, two studies were based on the same data set, and only one study (i.e., [2]) reported empirical evidence in support of the causal effect of job demands on teachers’ exhaustion.

Overall, since many previous longitudinal studies suffer from methodological limitations, there is no conclusive evidence for a general effect of job demands on exhaustion [25], or among teachers specifically [18]. Work pace and role conflict are reported to be job demands related to teachers’ exhaustion—and constitute prevalent examples of typical job demands within JD-R research [24, 25, 38]. Thus, the present study focusses on these job demands and use a three-wave design to investigate both causal and reversed causal effects between job demands and teachers’ exhaustion. Teachers are relevant for testing propositions in JD-R theory given that the teaching profession is a high-risk occupation for exhaustion–and an occupation suggested to be fairly standard with respect to the characteristics and the demands at work [12]. Thus, our study provides specific insights for the teaching profession as well as general insights in relation to JD-R theory and occupational health research.

### An expanded understanding of the effect of job demands on exhaustion—affective work rumination as a mediator

As teaching is a high-stress occupation, recovery and recuperation during non-work time has been suggested vital concern with respect to teachers’ work-related fatigue and exhaustion [1]. This suggestion is in line with current developments in stress theory, which emphasize the importance of attending to perseverative cognition (i.e., rumination and worry) for understanding the relationship between stress and ill health [39]. Perseverative cognition is suggested to increase the risk of ill health by prolonging the stress-related physiological activation of stressors and delaying or hindering recovery [40]. In a systematic review and meta-analysis, Clancy et al. [39] found that perseverative cognition in terms of rumination (but not reflection or worry) related to health risk behavior and suggested that rumination about stressors may mediate the effect of stressors on health outcomes.

Research on occupational health and well-being has predominately attended to this issue in terms of work rumination (i.e., when perseverative thinking occurs) and psychological detachment from work (i.e., when perseverative thinking does not occur). Work rumination refers to perseverative thoughts about work and work-related issues during non-work time, prolonging the stress-activation of stressors at work and hindering recovery [41]. When people experience high levels of job demands they tend to engage more in work ruminative thinking/be less psychologically detached [42–44].

Wendsche and Lohman-Haislah [45] argued that work rumination and psychological detachment from work can be understood as opposite ends of one dimension of mental distancing from work during non-work time, and that positive or negative emotions evoked by work seem to drive the relationship between work characteristics (e.g., job demands) and a tendency to engage in work ruminative thinking or being able to detach from work. Thus, a person who ruminates about work-related issues during non-work time is not psychologically detached from work, and vice versa [46]. However, Sonnentag and Fritz [43] reported correlations between psychological detachment from work and work rumination ranging between -.46 and -.49, suggesting that psychological detachment from work and work rumination are negatively related but not opposite ends. Nevertheless, only attending to the extent that people are (un)able to psychological detach from work may limit understanding, as it does not provide any information about what type of work-related thoughts a person tends to engage in [47]. Work rumination does not need to be negative, and when people think about work and work-related issues in a positive way, it may even contribute to recovery [48]. Furthermore, it has been reported [47, 49] that work-related rumination can be negative and maladaptive in terms of affective work rumination, or more positive and possibly constructive in terms of problem-solving pondering (i.e., unemotional, and solution-focused thinking).

The importance of differentiating, and specifying, the nature of work-related rumination that people may engage in is illustrated by reports demonstrating that affective work rumination is associated with more work-family conflict and less work-family enrichment, whereas problem-solving pondering is associated with more work-family enrichment [50]. Also, the results of a three-wave study found that whereas affective work rumination was associated with less off-job recovery, problem-solving pondering was associated with more creativity at work [51]. The importance of empirical and conceptual separation between psychological detachment and positive and negative work-related rumination has also been corroborated by the results of a recent meta-analyses. Comparing positive work-related thoughts and negative work-related thoughts, Jimenez et al. found that psychological detachment and problem-solving pondering only had rather weak relationships to exhaustion, whereas negative (and especially affective) work-related thoughts exhibited evident and stronger relationships [52].

Hence, regarding the health-impairment process as propositioned by JD-R theory, research suggests that affective work rumination is especially detrimental to employees’ ability to recover from stressors at work [53, 54]. Furthermore, reports indicate that psychological detachment from work and work rumination mediates the relationship between job demands and exhaustion by reducing or prolonging and accentuating the effect of job demands. Bennett et al. [29] examined the role of recovery experiences for the relationship between work characteristics (job demands and resources) and well-being (fatigue and vigor), as proposed by JD-R theory. The meta-analytical results showed that when recovery experiences were inserted as a partial mediator of the relationship between work characteristics (e.g., job demands) and well-being (e.g., fatigue), it contributed substantially to explaining the amount of variance compared to the models where work characteristics and recovery were treated as isolated single predictors. The results by Bennett et al. [29] need to be interpreted with some caution as the mediation analyses predominantly included relationships reported in cross-sectional studies, and predefined job demands based on the suggestions of the challenge-hindrance model of stress [55–57]. For instance, work pace was predefined as a challenge job demand whereas role conflict was predefined as a hindrance job demand–and the results showed that challenge job demands had a stronger negative effect on recovery compared to hindrance job demands. However, recent research suggests that this distinction is not substantiated [58], and that a possible differentiation in the effect of job demands may be better understood by attending to how workers appraise these work characteristics [59]. Yet, the results reported by Bennett et al. [29] suggest that to understand occupational well-being, at-work experiences (e.g., job demands) and after-work experiences (e.g., affective work rumination) should be given a joint focus. This suggestion is further substantiated by research reporting that affective work rumination mediates the association between emotion regulation strategies and exhaustion [60] stress and sleep disturbances [61], and between job demands and job performance, restoration, and sleep quality [62].

Given the results and reasoning above, the present study was designed to investigate causal and reciprocal relationships between job demands (i.e., work pace and role conflict) and exhaustion among elementary schoolteachers. Furthermore, the longitudinal three-wave design was set to test affective work-rumination as a mediator of the causal and reciprocal relationships between job demands and exhaustion. The three-wave design of the study was planned to collect data across a working year of Swedish schoolteachers (using three-month time-lags). For the present study we hypothesized that:

*Hypothesis 1a*: Work pace and role conflict (at T1 and T2) will have a positive cross-lagged effect on affective work rumination (at T2 and T3, respectively).
*Hypothesis 1b*: Affective work rumination (at T1 and T2) will have a positive cross-lagged effect on exhaustion (at T2 and T3, respectively).
*Hypothesis 2*: Affective work rumination (at T2) will *mediate* the positive cross-lagged effects of work pace and role conflict (T1) on exhaustion (T3).

However, in line with the JD-R proposition of reciprocal loss-cycles [17] and recent integration of a self-regulation perspective in the health-impairment process [23], exhaustion can determine future experiences of job demands. Furthermore, some research also reports results indicating a causal [30] and a reciprocal [47] relationship between exhaustion and recovery /work rumination [cf. 25].

Hence, we further hypothesized that:

*Hypothesis 3a*: Exhaustion (at T1 and T2) will have a positive cross-lagged effect on affective work rumination (at T2 and T3, respectively).
*Hypothesis 3b*: Affective work rumination (at T1 and T2) will have a positive cross-lagged effect on work pace and role conflict (at T2 and T3, respectively).
*Hypothesis 4*: Affective work rumination (at T2) will *mediate* the positive cross-lagged effect of exhaustion (T1) on work pace and role conflict (T3).

## Method

### Participants and procedure

The data was collected as part of a research project conducted on three professional groups in Sweden (teachers, psychologists, and ministers). For more information on the larger data collection and which construct what measured at the different time points please see S1 File. Data transparency statement and table. For teachers, initial contacts were made with representatives at the department for education at four municipalities geographically distributed across Sweden. Information about the study was given along with requests to receive email-addresses to all elementary school teachers employed in each municipality. In all, 7873 email addresses were collected.

The data collection used a three-wave design with three-month time lags. The time lags were based on the consideration of being able to investigate teachers’ variability in the study variables over a school year. In Sweden, teachers’ school year spans over one autumn semester (August—December) and one spring semester (from January—June). The waves of the data collection were planned so that any measurement occasion would not occur either too close to the start or the end of any semester (e.g., less/more demanding work periods). Thus, the three waves of the data collection were performed in late October 2017, late January 2018, and late April 2018.

Initial invitations to participate in the study were sent to 7873 teachers. To be eligible for participating the teacher should have worked at least one year in their profession. The emails contained information about the study and confidentiality, researchers’ contact information, and a link to the web-based survey. The survey was open for participation for three weeks, and two reminders were sent out (one per week). The survey took approximately 20 minutes to complete. All participants gave their informed consent. The responses were coded to ensure the highest amount of confidentiality and only one person in the project had access to the coding list.

In all, 1130 teachers responded to the invitation at Time 1. Out of these, 963 participants completed at least 75% of the questionnaire (12% participation rate). Four participants declined further participation (e.g., due to planned retirement/leave of absence), and two teachers who had missed the first survey wanted to participate in the two consecutive surveys. Thus, 961 teachers were invited at Time 2, and 705 responded (73% response rate) whereas 666 participants completed at least 75% of the questionnaire (69% participation rate). At Time 3, all 666 participating teachers from Time 2 were invited. Of these, 558 responded (84% response rate) and 525 completed at least 75% of the questionnaire (79% participation rate).

Attrition occurred, especially between Time 1 and Time 2. No differences were found in the reported levels of job demands, affective work rumination, or exhaustion between participants who participated across Time 1 –Time 3 and those participants who only participated at Time 1 and/or Time 2. However, slightly more men, χ^2^ (2, N = 1062) = 11.44, *p* = .002, and younger participants, *F*(2,1061) = 18.90, *p* <. 001, failed to answer the survey at Time 2 and Time 3. Following the recommendations of Newman [71], we included all participants who had answered at least one relevant construct at any time point and used full estimation maximum likelihood (FIML) in the main analyses. Thus, the final sample consisted of 1067 teachers. Of these 80% were women and the mean age was 47 years (SD age = 11 years). Regarding the sample’s representativeness, Sweden has approximately 100,900 elementary school teachers of which 75% are women and whose mean age is approximately 42 years [63].

### Materials

***Job demands*** were assessed in terms of work pace and role conflict, using the respective sub-dimensions on the validated Swedish medium-length version of the Copenhagen Psychosocial Questionnaire (COPSOQ II: [64, 65]). Work pace was measured by three items, rated on a five-point scale ranging from “never/almost never” (1) to “always” (5). Role conflict was measured by four items, rated on a five-point scale ranging from “to a very low degree” (1) to “to a very high degree” (5). Item examples are: “*Do you work at a high pace throughout the day*?” (work pace) and “*Are contradictory demands placed on you at work*?” (role conflict). For work pace, Cronbach’s alpha was .83 (T1), .84 (T2), and .84 (T3). For role conflict, Cronbach’s alpha was .68 (T1), .71 (T2), and .74 (T3).

***Affective work rumination*** was measured by the specific subscale on the Work Rumination Scale [66]. The subscale consists of five items, rated on a five-point Likert scale ranging from “very seldom” (1) to “very often/always” (5). An item example is: “*Do you become tense when you think about work-related issues during your free time*?”. Cronbach’s alpha was .92 (T1), .93 (T2), and .93 (T3).

***Exhaustion*** was assessed by the validated Swedish version of the Shirom Melamed Burnout Questionnaire (SMBQ: [67, 68]). The 22 items on the SMBQ are rated on seven-point scales, ranging from “almost never” (1) to “almost always” (7). An item example is: “*I feel exhausted*.” Cronbach’s alpha was .97 (T1-T3).

***Covariates*** included in the model were gender (male = 0, female = 1), age, working hours per week, and years working as a teacher.

### Data analyses

The data was analyzed by correlations using SPSS (version 25) and path analyses of cross-lagged effects in the three-wave full panel data using Mplus (version 8.2, [69]). The path analyses were based on full estimation maximum likelihood (FIML), which is pragmatic as it allows that all available information are used in the variables [70]. Furthermore, the path analysis was conducted in line with the recommendations for testing mediational models in three-wave full panel designs [71, 72].

## Results

### Descriptive statistics

The descriptive statistics and correlations between all study variables are reported in Table 1. Compared to the reference scores for work pace (*M* = 59.5) and role conflict (*M* = 42.5) obtained from the Danish working population [65], the mean levels in the present sample were higher. Furthermore, the reported mean levels of exhaustion were high (> 3.6) in comparison to the mean level observed (2.9) in the Swedish working population [73]. Significant and positively directed correlations were observed between all study variables and across the different points of measurement.

**Table 1:** Descriptive statistics and correlations (T1-T3).

	*N*	*M*	*SD*	*Skewness*	1	2	3	4	5	6	7	8	9	10	11	12
1. T1 Work pace	1065	73.50	16.54	-.37	**-**											
2. T1 Role conflict	1057	51.73	17.05	.03	.34	**-**										
3. T1 Affective work rumination	1044	3.13	.93	-.10	.38	.47	**-**									
4. T1 Exhaustion	938	3.80	1.31	.06	.33	.37	.70	^-^								
5. T2 Work pace	684	69.77	17.34	-.31	.70	.30	.30	.27	**-**							
6. T2 Role conflict	684	49.92	16.88	-.10	.32	.67	.44	.35	.37	**-**						
7. T2 Affective work rumination	677	3.00	.95	-.01	.39	.42	.72	.65	.41	.52	**-**					
8. T2 Exhaustion	660	3.63	1.28	.23	.30	.34	.57	.80	.31	.39	.72	**-**				
9. T3 Work pace	545	69.31	17.02	-.35	.69	.28	.31	.27	.75	.37	.39	.29	**-**			
10. T3 Role conflict	543	50.27	17.22	-.13	.33	.67	.46	.40	.36	.67	.46	.43	.38	**-**		
11. T3 Affective work rumination	540	2.97	.91	-.05	.40	.39	.74	.65	.38	.43	.78	.67	.41	.50	**-**	
12. T3 Exhaustion	519	3.59	1.34	.22	.33	.39	.60	.78	.33	.39	.66	.83	.34	.47	.71	**-**

Note. All correlations are significant at *p* < .001.

T1 = Time 1, T2 = Time 2, T3 = Time 3

### Tests of reciprocal model

To test the hypotheses the recommendations by Cole and Maxwell [71] for how to test mediation effects in longitudinal data was followed by sequentially building a reciprocal model. The authors specify which paths should be tested and their procedure is considered the gold standard for testing mediation effects. The testing starts with the building of a stability model to test the permanence in the measured variables across the adjacent time points. In this model, all exogenous variables were allowed to correlate, and all residuals of the endogenous variables were allowed to correlate within each time point. The model is then expanded include reciprocal effects by fitting cross-lagged effects of work pace and role conflict at T1 and T2 on affective work rumination at T2 and T3 respectively, as well as of affective work rumination at T1 and T2 on exhaustion at T2 and T3 respectively (i.e., causality effects). Then cross-lagged effects with paths from exhaustion at T1 and T) to affective work rumination at T2 and T3 respectively as well as the paths from affective work rumination at T1 and T2 to work pace and role conflict at T2 and T3, respectively (reversed causality effects) were added.

In accordance with the recommendation by Cole and Maxwell [71], the presence of non-linear effects among the study variables was tested by modeling higher order autoregressive effects. When these higher order autoregressive effects were added, specifying 2-lag direct effects of all variables between T1 and T3, the model fit was greatly improved which indicates that the studied variables display a quadratic effect over time. Thus, these effects were kept in the model.

Furthermore, to test if affective work rumination mediates the effect of work pace and role conflict at T1 on exhaustion at T3, direct effects between these variables were specified. Also, to investigate if affective work rumination mediates the respective effect of exhaustion on work pace and role conflict, direct effects of exhaustion at T1 on work pace and role conflict at T3 were fitted.

**Fig 1:**
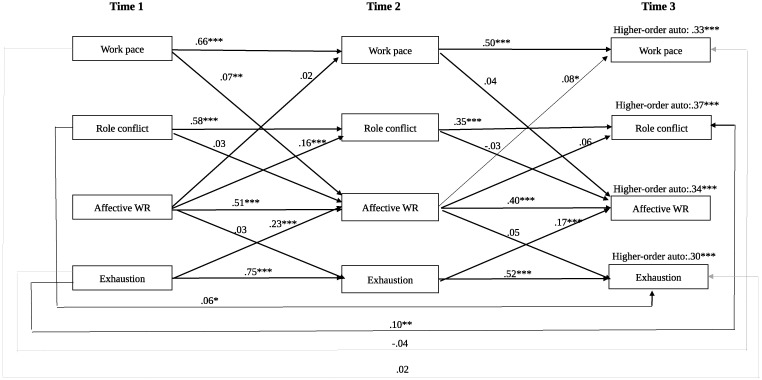
Crossed lagged standardizes regression weights for the final reciprocal model are presented. Solid arrows indicate significant paths and non-solid arrows indicated non-significant paths. Affective WR = Affective work rumination. Higher-order auto = Higher-order autocorrelations between Time 1 and Time 3. * p < .05, **p < .01, ***, p < .001.

The final reciprocal model had a good fit to data the χ^2^ (20) = 36.654 CFI = .997, TLI = 984, RMSEA .028 (.013-.042). As can be seen in Fig 1, *hypothesis 1a* was only partly confirmed since only work paces had a cross-lagged effect on work rumination, and *hypothesis 1b* was not confirmed since work rumination had no cross-lagged effect on exhaustion. However, *hypothesis 3a* was confirmed since exhaustion at T1 and T2 had a cross-lagged effect on work rumination at T2 and T3, respectively. But *hypothesis 3b* was only partly confirmed since work rumination at T1 only had a cross-lagged effect on role conflict at T2, and work rumination at T2 only had a cross-lagged effect on work pace at T3. The only reciprocal effect found was that between role conflict and exhaustion, where role conflict at T1 had an effect on exhaustion at T3, and exhaustion at T1 had an effect on role conflict at T3.

In the final model, when controlling for the other covariates the following results were observed. Females experienced a higher work pace (*β* = .15), more affective work rumination (*β* = .11), and more exhaustion (*β* = .10), at T1 (all *p* < .001). Older teachers experienced a lower work pace (*β* = -.16), less role conflict (*β* = -.13), less affective work rumination (*β* = -.14), and less exhaustion (*β* = -.19), at T1 (all *p* < .001). Teachers who had worked more years in their profession experienced higher work pace (*β* = .11), at T1 (*p* = .001). Also, teachers who worked more hours per week experienced a higher work pace (*β* = .18), more role conflict (*β* = .08), and more work rumination (*β* = .10), at T1 (all *p* < .01). No effects of the covariates were found on the variables at T2 and T3.

To test mediation effects, indirect effects were calculated using bootstrapped estimation of standard errors. No support was found for *hypothesis 2* since the indirect effects of work pace and role conflict (T1) on exhaustion (T3), via affective work rumination (T2), were non-significant (*p* >.05). Also, the indirect effect of exhaustion (T1) on role conflict (T3), via affective work rumination (T2), was non-significant (*p* > .05). However, there was a small effect of exhaustion (T1) to work pace (T3), via affective work rumination (T2) (β = 0.02, SE = 0.009, *p* = .03), which lend modest support to *hypothesis 4*.

### Alternative model

Instead of affective work rumination being the mediator it could be argued that exhaustion may be directly related to experiences of higher work pace and role conflict, since exhaustion is associated with various types of self-undermining behavior and increased levels of job demands [20, 21]. In turn, experiences of higher levels of demands may lead to higher levels of affective work rumination, which instead make job demands the mediator. Based on this reasoning, an alternative model was tested were job demands (i.e., work pace and role conflict) mediate the relationship between exhaustion and affective work rumination. The alternative model was fitted by placing cross-lagged paths from exhaustion at T1 and T2 to work pace and role conflict at T2 and T3, respectively. Paths were also fitted from work pace and role conflict atT1 and T2 to affective work rumination at T2 and T3 respectively. Also, 2-lag autoregressive effects between T1 and T3 for all variables were fitted.

As can be seen in S1 Fig, exhaustion at T1 and T2 was found to predict role conflict at T2 and T3 respectively but did not predict work pace. The support for job demands predicting affective work rumination was scarce since the only significant path was observed between work pace at T1 and affective work rumination at T2. Thus, job demands were not found to mediate the relationship between exhaustion and affective work rumination. However, there was a strong direct effect between exhaustion at T1 and affective work rumination at T3. The model fit was adequate, χ^2^ (27) = 131.247, CFI = .983, TLI = 927, RMSEA .060 (.050-.071), but worse than the model fit of the reciprocal model reported above, delta χ^2^ (7) = 94.593, *p* < .001.

## Discussion

This study investigated causal effects and reciprocal relationships between job demands and exhaustion among teachers [18]. The study also aimed to contribute to JD-R theory by examining if affective work rumination mediates the relationships between job demands and exhaustion. Specifically, we examined if affective work rumination is a psychological mechanism (i.e., maladaptive cognition) that contributes to the explanation of the job demands—exhaustion relationship [17, 23]. Overall, the results provided partial empirical evidence for the hypothesized cross-lagged effects and reciprocal relationships, but only modest support for the hypotheses that affective work rumination mediate these effects.

In the tested model, work pace (T1) was found to influence near-future affective work rumination (i.e., at T2, three months later). Hence, the evidenced causal effect suggests that when teachers experience higher work pace, it triggers the tendency to engage in negative rumination about work during non-work time and evoke associated feelings of frustration and irritability (hypothesis 1a).

Furthermore, the tested model provided support for the notion that when teachers experience tasks and requirements at work as unclear and contradictory (i.e., role conflict) at the beginning of a schoolyear, it affects levels of exhaustion at the end of the schoolyear. Also, our results indicate that engaging in affective work rumination influences teachers’ experiences of future job demands (hypothesis 3b). This result supports the notion that maladaptive cognitions can foster, or increase, future experiences of job demands (*Proposition 8* in JD-R theory). However, the results only provided partly support for these effects (i.e., affective work rumination at T1 affected role conflict at T2, whereas affective work rumination at T2 affected work pace at T3).

The effect of exhaustion on affective work rumination (hypothesis 3b) was evident since cross-lagged associations were observed at both time sequences (i.e., T1 –T2 and T2 –T3). This substantiates previous research [30], by demonstrating that exhausted employees appear more inclined to engage in work rumination (cf. report being less psychologically detached from work). Furthermore, the results showed that teachers who report tendencies toward exhaustion are more prone to experience higher future levels of role conflicts. In fact, the results demonstrated support for a reciprocal relationship between role conflict and exhaustion. Moreover, modest support was observed for the hypothesis that affective work rumination act as a mediator of the job demands–exhaustion relationship (hypothesis 4), as affective work rumination (at T2) was found to partially mediate the effect of exhaustion (at T1) on work pace (at T3).

Overall, our results indicate that the prevailing assumption that job demands are the main determinant of teachers’ exhaustion needs to be further elaborated on. Though high levels of work pace and role conflict can be problematic for teachers, our results did not show that the effect of job demands on teachers’ exhaustion was evident. However, in line with recent reports [24, 25], the model provided clearer support for the reversed and reciprocal relationships between job demands and exhaustion, as compared to the basic causal effects.

### Contribution to JD-R theory

Our study contributes to the limited number of studies that have tested the causal and the reciprocal assumptions of JD-R theory [20, 24, 25]. In fact, previous studies that have examined the basic assumption of JD-R theory across more than two intervals of measurement are rather few (see Table 2 in Lesener et al., [24]). In line with recent recommendations and suggestions [24, 25], our study makes further contributions by the use of a three-wave design and time-lags shorter than one year (and of theoretical and methodological relevance for the specific study/sample), addressing reciprocal relationships, and examining the role of recovery in the stressor-strain relationship.

The results of our study illustrates the need to examine the role and importance of specific job demands within particular occupational groups and/or professions [38], since the effects and relationships between job demands and exhaustion may be rather unique [23, 31]. We found a reciprocal relationship between role conflict and exhaustion, and the results further indicate that teachers who experience tendencies to exhaustion will be more likely to engage in future affective work rumination. These results underline the importance of exhaustion at baseline for understanding the development of occupational health and well-being [74]. Exhaustion is a negative affective state associated with pessimistic thinking (cf. disengagement/cynicism: [75]). Our study corroborates this association by demonstrating reciprocity between exhaustion and an inclination to doubt the purpose and meaning of tasks and requirements at work (role conflict). Our results also indicate that exhaustion instigates maladaptive thoughts and emotions with respect to work in terms of affective work rumination. This result provides empirical support for the JD-R propositions of loss-cycles [20] and the integration of a self-regulation perspective [23], by showing that teachers who experience higher levels of exhaustion are more inclined to engage in maladaptive cognitions (i.e., affective work rumination) and to experience higher future levels of role conflict at work. That is, our results support the notion of a loss cycle starting from exhaustion to self-undermining (i.e., maladaptive cognition in terms of affective work rumination) and further on to experienced job demands.

However, our results do not support that job demands instigate the health-impairment process as precursors for work ruminative tendencies [45, 46]. This means that our study does not corroborate the report by Bennett et al. [29] that recovery experiences mediate the JD-R propositioned association between work characteristics (e.g., job demands) and well-being (e.g., exhaustion). Neither do our results support the idea that work rumination prolong the effect of job demands or that the interplay between job demands and work rumination explains exhaustion among teachers [41]. Concerning these divergent results, we examined work rumination as a mediator based on longitudinal data observations whereas the reports by Cropley and Purvis [41] and (predominantly also) by Bennett et al. [29] were based on observations of cross-sectional data.

In brief, our results provide empirical for certain propositions in JD-R theory [18, 20]. The results add some support for *proposition 2* (i.e., job demands instigate a health-impairment process), *proposition 8* (i.e., job demands and strain may lead to maladaptive cognition), and *proposition 9* (i.e., job strain can initiate a loss-cycle of maladaptive cognition and job demands) [76]. Furthermore, our study contributes by providing insights regarding the role of affective work rumination as an aspect of maladaptive cognition relevant for understanding loss-cycle relationships [25].

### Practical implications

Our results support that work pace and role conflict are prominent demands in the teaching profession [4, 5, 7], and show that role conflict seems to be especially important for understanding teachers’ exhaustion. Arguably, the prominence and importance of role conflict can be understood in relation to increase in administrative tasks and accountability of the teacher profession [71, 72]. However, the reciprocal relationship between exhaustion and role conflict found in our study suggests that neither of these constructs can be considered solely as a cause or a consequence. Teachers who experience high levels of role conflict may take on more tasks as they may have trouble finding their professional boundaries which, over time, may lead to exhaustion. Moreover, teachers who already experience exhaustion may not be able to guard their professional boundaries and (for example as an effort to avoid conflict with colleagues, parents, and students) may be more inclined to take on further obligations and tasks which in turn leads to higher exhaustion (i.e., initiating a loss cycle [17, 20, 22, 23, 76, cf. 77, 78]). Thus, when addressing exhaustion among teachers, obligations of the professional role need specific attention. Accordingly, one way to improve teachers’ occupational health and well-being is to lower the work pace and to address the causes for experiences of role conflict. However, proactive stress preventions is another (or complementary) way to go in order to improve the situation for teachers.

Our results further suggest that to break the vicious spiral between high job demands and exhaustion it would be helpful to facilitate the possibility for teachers to recover or to provide support to prevent affective work rumination. This could include helping teachers to finish more on-going tasks at the end of the workday or workweek since this is likely to decrease their affective work rumination and improve their restorative rest before the next work occasion. The results show that this is of importance for teachers who experience high levels of job demands, and for teachers who have started to develop tendencies toward exhaustion. Although some intervention studies have investigated how recovery may be improved and tendencies to burnout reduced [79], our understanding is still rather limited regarding how organizations can promote recovery processes–at work or after work (for a discussion, see [46, 80]). Nevertheless, in relation to the challenging conditions for teachers, our results underline the importance of finding ways to reduce teachers’ work pace (and the associated workload) and to provide assistance. In addition, improved clarity in relation to the professional requirements would be helpful, that is to refine the role of teachers [14, 15]. Arguably, improving the conditions for teachers also requires commitment on the political and the organizational level. Such commitment would be justified, as improving the conditions for teachers can be expected to have positive pedagogical, educational, and, ultimately, societal effects.

### Limitations and recommendations for future research

Our study has some limitations. We used a three-wave design with three-month time lags. These time lags were considered relevant for the study/sample (i.e., capturing relationships among teachers across a schoolyear). Thus, in line with recent recommendations, the time lags of the present study were clearly motivated [24] and shorter than one year [25]. The observed relationships between affective work rumination and exhaustion in our study to some extent agree with previous reports that have used six-months [53] or one year [47] time lags. However, it may be that three-month time lags are not optimal for testing if affective work rumination mediates the stressor–strain relationship. Thus, recovery experiences and/or negative work rumination could be of importance for relationships between job demands and exhaustion at other time lags. Longitudinal studies investigating the role of recovery (e.g., affective work rumination) in the link between short-term (e.g., experiences of demands at work) and long-term processes (e.g., exhaustion) have been called for [23, 46, 82]. Further research could investigate different time intervals to specify optimal time lags since the length of time intervals impacts statistical power [81].

Moreover, although the measurement of the predictor variables, the mediator, and the criterion variable were temporally separated and the base-line levels controlled for in the analyses (as required to analyze mediation: [72]), our study is based on self-reporting. Therefor the results should be interpreted with potential common method bias in mind [82]. Yet, it could be asked if it is feasible to obtain measurements from other sources to assess subjective experiences of work pace, role conflict, affective work rumination, and/or exhaustion [82].

The rather low response rate at Time 1 (14%) may be considered as a limitation. As the invitations to participate in the study informed about the three-wave design, this may have reduced the level of initial participation and is a general problem for longitudinal studies. The present sample predominately consisted of female teachers (80%) and some previous research has found that women tend to engage in more ruminative thinking compared to men [83]. Even though the present sample is representative of the teacher population in Sweden (75% women: [63]), future research should investigate possible gender differences regarding the role of work rumination in occupational health and well-being.

From the current theoretical standpoint, work rumination seems most likely to be regarded as a mediator of the job demands–exhaustion relationship [23, 29, 76]. Given the focus on the health-impairment process, the present study only examined the mediating role of affective work rumination. We encourage future research to take a broader approach by including and investigating different aspects of work-related rumination (i.e., affective work rumination, psychological detachment, and problem-solving pondering [28, 47, 53]) as possible mediators of causal and reciprocal relationships between job demands and exhaustion. Broader approaches could also include job resources as well as various types of outcomes (e.g., work engagement), and investigate possible loss cycle and gain cycle relationships [76]. Such investigations could offer further insights to encompass the role of recovery and work-related rumination in JD-R theory.

## Conclusions

This study adds to the rather limited longitudinal research on one of the basic propositions in JD-R theory, that job demands trigger the health-impairment process [17, 20]. Furthermore, our study contributes by investigating relationships between job demands and exhaustion in a large sample of teachers by means of a longitudinal design, for which previous reports of associations mainly have been based on cross-sectional studies [18]. Interestingly, our results only provided limited support for a direct effect of job demands (i.e., role conflict) on teachers’ future exhaustion.

Corresponding to current theoretical developments and recent meta-analytical results [23–25, 29, 76], we investigated the role of work rumination in causal and reciprocal relationships between job demands and exhaustion. Our results demonstrated that the reciprocal model had the best fit to the data, and a reciprocal relationship was found between role conflict and exhaustion. Exhaustion was found to be related to accumulated future levels of affective work rumination. However, the results did only provide partial support for the mediating effect of affective work rumination for the job demands and exhaustion relationship. In summary, further investigations should attend to the role of different types of work ruminative thinking in the health-impairment process, in general and among teachers specifically. Such investigations offer one interesting line of enquiry for further developments of JD-R theory.

## Supporting information

S1 File Data transparency statement and table. (DOCX)

S1 Fig Alternative model. (DOCX)

## References

[1] Garrick A, Mak AS, Cathcart S, Winwood PC, Bakker AB, Lushington K. Non-work time activities predicting teachers’ work-related fatigue and engagement: An effort-recovery approach. Austral Psychol 2018 Jun 1;53(3):243–52.

[2] Prieto LL, Soria MS, Martínez IM, Schaufeli W. Extension of the Job Demands-Resources model in the prediction of burnout and engagement among teachers over time. Psicothema 2008 Dec 31:354–60. 18674427

[3] Salmela-Aro K, Hietajärvi L, Lonka K. Work burnout and engagement profiles among teachers. Front Psychol 2019 Oct 4;10:2254. 31636591 10.3389/fpsyg.2019.02254 PMC6787897

[4] Skaalvik EM, Skaalvik S. Job demands and job resources as predictors of teacher motivation and well-being. Soc Psychol Educ 2018 Nov;21(5):1251–75.

[5] Arvidsson I, Håkansson C, Karlson B, Björk J, Persson R. Burnout among Swedish school teachers–a cross-sectional analysis. BMC Public Health 2016 Dec;16(1):1–1. 27539073 10.1186/s12889-016-3498-7 PMC4991104

[6] Hakanen JJ, Bakker AB, Schaufeli WB. Burnout and work engagement among teachers. J School Psychol 2006 Jan 1;43(6):495–513.

[7] Skaalvik EM, Skaalvik S. Dimensions of teacher burnout: Relations with potential stressors at school. Soc Psychol Educ 2017 Dec;20:775–90.

[8] Fernet C, Chanal J, Guay F. What fuels the fire: Job- or task-specific motivation (or both)? On the hierarchical and multidimensional nature of teacher motivation in relation to job burnout. Work & Stress 2017;31:145–63.

[9] Swedish Teachers’ Union (2016). Let teachers be teachers. Perspectives on the teaching profession–workload in the elementary school. The Swedish Teachers’ Union. Stockholm, Sweden; 2016.

[10] Swedish National Agency for Education (2016). Attitudes to the school 2015. Report 438. The Swedish National Agency for Education. Stockholm, Sweden; 2016.

[11] Swedish Teachers’ Union (2017). Not enough time–review of workload and sick-leave among teachers. The Swedish Teachers’ Union, Stockholm, Sweden; 2017.

[12] Swedish Teachers’ Union (2018). Stuck in an unbalance between demands and resources–review of teachers’ workload and stress. The Swedish Teachers’ Union, Stockholm, Sweden; 2018.

[13] Swedish National Agency for Education (2019). Teacher prognosis 2019. Dnr. 5.1.3–2018:1500. The Swedish National Agency for Education, Stockholm, Sweden; 2019.

[14] Statistics Sweden (2023). Teachers outside the profession 2022/2023. Report 2023–6. Statistics Sweden, Stockholm, Sweden; 2023.

[15] Swedish National Agency for Education (2018). Teacher supply. Dnr. 2018:00234. The Swedish National Agency for Education, Stockholm, Sweden; 2018.

[16] Bakker AB, Demerouti E. Job Demands-Resources theory. Work and well-being: A complete reference guide, vol. III. In Chen PY & Cooper CL, editors. Work and well-being: A complete reference guide. Chichester, UK: John Wiley and Sons, Ltd.; 2014. p. 3–28.

[17] Bakker AB, Demerouti E. Job demands–resources theory: Taking stock and looking forward. J Occup Health Psychol 2017 Jul;22(3):273. 27732008 10.1037/ocp0000056

[18] Taris TW, Leisink PLM, Schaufeli WB. Applying occupational health theories to educator stress: Contribution of the Job Demands-Resources Model. In: McIntyre TM, McIntyre SE, Francis DJ, editors. Educator stress. Cham: Springer International Publishing; 2017, p. 237–59.

[19] Demerouti E, Bakker AB, Nachreiner F, Schaufeli WB. The job demands-resources model of burnout. J Appl Psychol 2001 Jun;86(3):499. 11419809

[20] Bakker AB, Demerouti E. Multiple levels in job demands-resources theory: Implications for employee well-being and performance. In: Diener E, Oishi S, & Tay L, editors. Handbook of well-being. Salt Lake City, UT: DEF Publishers.2018(2018):1–3.

[21] Bakker AB, Costa PL. Chronic job burnout and daily functioning: A theoretical analysis. Burnout research 2014 Dec 1;1(3):112–9.

[22] ten Brummelhuis LL, Ter Hoeven CL, Bakker AB, Peper B. Breaking through the loss cycle of burnout: The role of motivation. J Occup Org Psychol 2011 Jun;84(2):268–87.

[23] Bakker AB, de Vries JD. Job Demands–Resources theory and self-regulation: New explanations and remedies for job burnout. Anxiety, Stress, & Coping 2021 Jan 2;34(1):1–21. 32856957 10.1080/10615806.2020.1797695

[24] Lesener T, Gusy B, Wolter C. The job demands-resources model: A meta-analytic review of longitudinal studies. Work & Stress 2019 Jan 2;33(1):76–103.

[25] Guthier C, Dormann C, Voelkle MC. Reciprocal effects between job stressors and burnout: A continuous time meta-analysis of longitudinal studies. Psych Bul 2020 Dec;146(12):1146. 10.1037/bul0000304 33119345

[26] Tang K. A reciprocal interplay between psychosocial job stressors and worker well-being? A systematic review of the" reversed" effect. Scand J Work, Environ & Health 2014 Sep 1:441–56. 10.5271/sjweh.3431 24756578

[27] Casper A, Sonnentag S. Feeling exhausted or vigorous in anticipation of high workload? The role of worry and planning during the evening. J Occup Org Psychol 2020 Mar;93(1):215–42.

[28] Firoozabadi A, Uitdewilligen S, Zijlstra FR. Solving problems or seeing troubles? A day-level study on the consequences of thinking about work on recovery and well-being, and the moderating role of self-regulation. Europ J Work Org Psychol 2018 Sep 3;27(5):629–41.

[29] Bennett AA, Bakker AB, Field JG. Recovery from work-related effort: A meta-analysis. J Org Behav 2018 Mar;39(3):262–75.

[30] Sonnentag S, Arbeus H, Mahn C, Fritz C. Exhaustion and lack of psychological detachment from work during off-job time: moderator effects of time pressure and leisure experiences. J Occup Health Psychol 2014 Apr;19(2):206. 24635737 10.1037/a0035760

[31] Toppinen-Tanner S, Kalimo R, Mutanen P. The process of burnout in white-collar and blue-collar jobs: eight-year prospective study of exhaustion. J Org Behav 2002 Aug;23(5):555–70.

[32] Barbieri B, Sulis I, Porcu M, Toland MD. Italian teachers’ well-being within the high school context: evidence from a large scale survey. Front Psychol 2019 Aug 21;10:1926. 31496981 10.3389/fpsyg.2019.01926 PMC6713017

[33] Fernet C, Austin S, Trépanier SG, Dussault M. How do job characteristics contribute to burnout? Exploring the distinct mediating roles of perceived autonomy, competence, and relatedness. Europ J Work Org Psychol 2013 Apr 1;22(2):123–37.

[34] Pyhältö K, Pietarinen J, Salmela-Aro K. Teacher–working-environment fit as a framework for burnout experienced by Finnish teachers. Teach Teacher Educ 2011 Oct 1;27(7):1101–10.

[35] Skaalvik EM, Skaalvik S. Teacher self-efficacy and teacher burnout: A study of relations. Teach Teacher Educ 2010 May 1;26(4):1059–69.

[36] Dicke T, Stebner F, Linninger C, Kunter M, Leutner D. A longitudinal study of teachers’ occupational well-being: Applying the job demands-resources model. J Occup Health Psychol 2018 Apr;23(2):262–77. 28150993 10.1037/ocp0000070

[37] González-Morales MG, Rodríguez I, Peiró JM. A longitudinal study of coping and gender in a female-dominated occupation: Predicting teachers’ burnout. J Occup Health Psychol 2010 Jan;15(1):29. 20063957 10.1037/a0018232

[38] Schaufeli WB, Taris TW. A critical review of the Job Demands-Resources Model: Implications for improving work and health. Bridging occupational, organizational and public health. Dordrecht: Springer Netherlands; 2014. p. 43–68.

[39] Clancy F, Prestwich A, Caperon L, O’Connor DB. Perseverative cognition and health behaviors: A systematic review and meta-analysis. Front Human Neurosc 2016 Nov 8;10:534. 10.3389/fnhum.2016.00534 PMC5099163 27877119

[40] Brosschot JF, Gerin W, Thayer JF. The perseverative cognition hypothesis: A review of worry, prolonged stress-related physiological activation, and health. J Psychosom Research 2006 Feb 1;60(2):113–24. 16439263 10.1016/j.jpsychores.2005.06.074

[41] Cropley M, Millward Purvis L. Job strain and rumination about work issues during leisure time: A diary study. Europ J Work Org Psychol 2003 Sep 1;12(3):195–207.

[42] Sonnentag S, Binnewies C, Mojza EJ. Staying well and engaged when demands are high: the role of psychological detachment. J Appl Psychol 2010 Sep;95(5):965. 20718528 10.1037/a0020032

[43] Sonnentag S, Fritz C. Recovery from job stress: The stressor-detachment model as an integrative framework. J Org Beh 2015 Feb;36(S1):S72–103.

[44] Cropley M, Dijk DJ, Stanley N. Job strain, work rumination, and sleep in school teachers. Europ J Work Org Psychol 2006 Jun 1;15(2):181–96.

[45] Wendsche J, Lohmann-Haislah A. A meta-analysis on antecedents and outcomes of detachment from work. Front Psychol 2017 Jan 13;7:2072. 28133454 10.3389/fpsyg.2016.02072 PMC5233687

[46] Sonnentag S. The recovery paradox: Portraying the complex interplay between job stressors, lack of recovery, and poor well-being. Research Org Beh 2018 Jan 1;38:169–85.

[47] Kinnunen U, Feldt T, de Bloom J. Testing cross-lagged relationships between work-related rumination and well-being at work in a three-wave longitudinal study across 1 and 2 years. J Occup Org Psychol 2019 Sep;92(3):645–70.

[48] Demerouti E, Bakker AB, Geurts SAE, Taris TW. Daily recovery from work-related effort during non-work time. In: Sonnentag S, Perrewé PL, Ganster DC, editors. Research in occupational stress and well-being, vol. 7. Bingley, UK: Emerald Group; 2009, p. 85–123.

[49] Cropley M, Zijlstra F. Work and rumination. In: J Langan-Fox J, & Cooper CL, editors. Handbook of stress in the occupations. Northampton, MA: Edward Elgar Publishing Ltd; 2011, p. 487–502.

[50] Junker NM, Baumeister RF, Straub K, Greenhaus JH. When forgetting what happened at work matters: The role of affective rumination, problem-solving pondering, and self-control in work–family conflict and enrichment. J Appl Psychol 2021 Nov;106(11):1750. 33090861 10.1037/apl0000847

[51] Vahle-Hinz T, Mauno S, De Bloom J, Kinnunen U. Rumination for innovation? Analysing the longitudinal effects of work-related rumination on creativity at work and off-job recovery. Work & Stress 2017 Oct 2;31(4):315–37.

[52] Jimenez WP, Hu X, Xu XV. Thinking about thinking about work: A meta-analysis of off-job positive and negative work-related thoughts. J Bus Psychol 2022 Apr;37(2):237–62.

[53] Firoozabadi A, Uitdewilligen S, Zijlstra FR. Should you switch off or stay engaged? The consequences of thinking about work on the trajectory of psychological well-being over time. J Occup Health Psychol 2018 Apr;23(2):278–88. 27991803 10.1037/ocp0000068

[54] Querstret D, Cropley M. Exploring the relationship between work-related rumination, sleep quality, and work-related fatigue. J Occup Health Psychol 2012 Jul;17(3):341. 22746369 10.1037/a0028552

[55] Cavanaugh MA, Boswell WR, Roehling MV, Boudreau JW. An empirical examination of self-reported work stress among US managers. J Appl Psychol 2000 Feb;85(1):65. 10740957 10.1037/0021-9010.85.1.65

[56] Crawford ER, LePine JA, Rich BL. Linking job demands and resources to employee engagement and burnout: a theoretical extension and meta-analytic test. J Appl Psychol 2010 Sep;95(5):834–48. 20836586 10.1037/a0019364

[57] LePine JA, Podsakoff NP, LePine MA. A meta-analytic test of the challenge stressor–hindrance stressor framework: An explanation for inconsistent relationships among stressors and performance. AMJ 2005 Oct 1;48(5):764–75.

[58] Mazzola JJ, Disselhorst R. Should we be “challenging” employees?: A critical review and meta-analysis of the challenge-hindrance model of stress. J Organ Behav 2019 Oct;40(8):949–61.

[59] Li P, Taris TW, Peeters MC. Challenge and hindrance appraisals of job demands: one man’s meat, another man’s poison? Anxiety, Stress, & Coping. 2020 Jan 2;33(1):31–46. 31578098 10.1080/10615806.2019.1673133

[60] Geisler M, Buratti S, Allwood CM. The Complex Interplay Between Emotion Regulation and Work Rumination on Exhaustion. Front Psychol 2019;10:1978. doi: 10.3389/fpsyg.2019.01978 31555174 PMC6727428

[61] Van Laethem M, Beckers DG, van Hooff ML, Dijksterhuis A, Geurts SA. Day-to-day relations between stress and sleep and the mediating role of perseverative cognition. Sleep Medicine 2016 Aug 1;24:71–9. 27810189 10.1016/j.sleep.2016.06.020

[62] Van Laethem M, Beckers DG, de Bloom J, Sianoja M, Kinnunen U. Challenge and hindrance demands in relation to self-reported job performance and the role of restoration, sleep quality, and affective rumination. J Occup Org Psychol 2019 Jun;92(2):225–54.

[63] Statistics Sweden. The Swedish occupational register with statistics 2016. Stockholm: Statistics Sweden. 2018.

[64] Berthelsen H, Hakanen JJ, Westerlund H. Copenhagen psychosocial questionnaire-a validation study using the job demand-resources model. PloS one 2018 Apr 30;13(4):e0196450. 29708998 10.1371/journal.pone.0196450 PMC5927437

[65] Pejtersen JH, Kristensen TS, Borg V, Bjorner JB. The second version of the Copenhagen Psychosocial Questionnaire. Scand J Public Health 2010 Feb;38(3_suppl):8–24. 21172767 10.1177/1403494809349858

[66] Cropley M, Michalianou G, Pravettoni G, Millward LJ. The relation of post-work ruminative thinking with eating behaviour. Stress and Health 2012 Feb;28(1):23–30. 22259155 10.1002/smi.1397

[67] Lundgren-Nilsson Å, Jonsdottir IH, Pallant J, Ahlborg G. Internal construct validity of the Shirom-Melamed burnout questionnaire (SMBQ). BMC Public Health 2012 Dec;12:1–8. 22214479 10.1186/1471-2458-12-1 PMC3307433

[68] Melamed S, Kushnir T, Shirom A. Burnout and risk factors for cardiovascular diseases. Behav Med 1992 Jun 1;18(2):53–60. 1392214 10.1080/08964289.1992.9935172

[69] Muthén LK, Muthén B. Mplus user’s guide: Statistical analysis with latent variables, user’s guide. Muthén & Muthén; 2017.

[70] Newman DA. Missing data: Five practical guidelines. Org Research Meth 2014 Oct;17(4):372–411.

[71] Cole DA, Maxwell SE. Testing mediational models with longitudinal data: questions and tips in the use of structural equation modeling. J Abnor Psychol 2003 Nov;112(4):558. 10.1037/0021-843X.112.4.558 14674869

[72] Taris TW, Kompier MA. Games researchers play—Extreme-groups analysis and mediation analysis in longitudinal occupational health research. Scand J Work Environ Health 2006 Dec 1:463–72. 10.5271/sjweh.1051 17173202

[73] Norlund S, Reuterwall C, Höög J, Lindahl B, Janlert U, Birgander LS. Burnout, working conditions and gender-results from the northern Sweden MONICA Study. BMC Public Health 2010 Dec;10(1):1–9. 20534136 10.1186/1471-2458-10-326 PMC2896942

[74] Michielsen HJ, Willemsen TM, Croon MA, De Vries J, Van Heck GL. Determinants of general fatigue and emotional exhaustion: A prospective study. Psychol & Health 2004 Apr 1;19(2):223–35.

[75] Maslach C, Schaufeli WB, Leiter MP. Job burnout. Annu Rev Psychol 2001 Feb;52(1):397–422. 11148311 10.1146/annurev.psych.52.1.397

[76] Bakker AB, Demerouti E, Sanz-Vergel A. Job Demands-Resource theory: ten years later. Annu. Rev. Organ. Psychol. Organ. Behav 2023; 10:25–53.

[77] Hobfoll SE. Conservation of resources: A new attempt at conceptualizing stress. American Psychologist 1989; 44(3), 513 2648906 10.1037//0003-066x.44.3.513

[78] Hobfoll SE. The influence of culture, community, and the nested-self in the stress process: Advancing conservation of resources theory. Applied Psychology 2001; 50(3): 337–421.

[79] Siu OL, Cooper CL, Phillips DR. Intervention studies on enhancing work well-being, reducing burnout, and improving recovery experiences among Hong Kong health care workers and teachers. Int J Stress Manag 2014 Feb;21(1):69.

[80] Sonnentag S, Cheng BH, Parker SL. Recovery from work: Advancing the field toward the future. Annu Rev Org Psychol Org Behav 2022 Jan 21;9:33–60.

[81] Dormann C, Griffin MA. Optimal time lags in panel studies. Psychol Meth 2015 Dec;20(4):489. 10.1037/met0000041 26322999

[82] Podsakoff PM, MacKenzie SB, Lee JY, Podsakoff NP. Common method biases in behavioral research: a critical review of the literature and recommended remedies. J Appl Psychol 2003 Oct;88(5):879–903. 14516251 10.1037/0021-9010.88.5.879

[83] Jose PE, Brown I. When does the gender difference in rumination begin? Gender and age differences in the use of rumination by adolescents. J Youth Adolesc 2008 Feb;37:180–92.

