# Supplementary material for: Affective work rumination as a mediator of the reciprocal relationships between job demands and exhaustion
Source: PLoS One. 2023 Nov 9;18(11):e0293837. doi: 10.1371/journal.pone.0293837 (PMC10635451; doi:10.1371/journal.pone.0293837)
Supplement: S1 File — (DOCX) [file pone.0293837.s001.docx]

**Data transparency statement and table.**

The data reported in the current manuscript (MS 4) was collected as part of a larger data collection (at one or more points in time). The larger data collection consisted of a larger survey that was distributed at three time points to the professional groups at each time point (priests, teachers and clinical psychologists). For more details of which psychological constructs that was measured at which time point please see table below.

Findings from the larger data collection have been reported in separate manuscripts. MS 1 (published) included participants from all three professional groups studied and focuses on variables 7, 10, and 19 (at T1). MS 2 (published) specifically addressed data collected from clinical psychologist, and focuses on variables 1-4, 6-7, 9, 11, 15, and 18-19 (at T1). MS 3 (published) only addressed data collected from priests, and focuses on variables 1, 3-5, 9, 11, 15, and 18-19 (at T1). MS 4 (the current manuscript) specifically address data collected from teachers, and focuses on variables 1, 6-7, and 19 (at T1-T3).

The table below reports information for the data collection and displays where each data variable appears in each study.

| **Complete data set** | **Data wave** | **MS 1**  **published** | **MS 2**  **published** | **MS 3**  **published** | **MS 4**  **submitted** |
| --- | --- | --- | --- | --- | --- |
| *Teachers* | T1-T3 | X |  |  | X |
| *Clinical psychologists* | T1-T3 | X | X |  |  |
| *Priests* | T1-T3 | X |  | X |  |
| Data wave | - | T1 | T1 | T1 | T1-T3 |
| 1. Gender | T1 |  | X | X |  |
| 1. Work pace | T1-T3 |  | X |  | X |
| 1. Quantitative demands | T1-T3 |  | X | X |  |
| 1. Emotional demands | T1-T3 |  | X | X |  |
| 1. Role clarity | T1-T3 |  |  | X |  |
| 1. Role conflict | T1-T3 |  | X |  | X |
| 1. Work rumination | T1-T3 | X | X |  | X |
| 1. Brooding | T1-T3 |  |  |  |  |
| 1. Personal to work conflict | T1-T3 |  | X | X |  |
| 1. Emotion regulation | T2-T3 | X |  |  |  |
| 1. Prosocialness | T1-T3 |  | X | X |  |
| 1. Stress of consciousness | T2-T3 |  |  |  |  |
| 1. Proactive attitude scale | T1-T3 |  |  |  |  |
| 1. Decision-making styles | T1-T3 |  |  |  |  |
| 1. Relational-interdependent self-construal | T1-T3 |  | X | X |  |
| 1. Work ability | T1-T3 |  |  |  |  |
| 1. Perceived stress | T1-T3 |  |  |  |  |
| 1. Disengagement | T1-T3 |  | X | X |  |
| 1. Exhaustion | T1-T3 | X | X | X | X |
