# Supplementary material for: Affective work rumination as a mediator of the reciprocal relationships between job demands and exhaustion
Source: PLoS One. 2023 Nov 9;18(11):e0293837. doi: 10.1371/journal.pone.0293837 (PMC10635451; doi:10.1371/journal.pone.0293837)
Supplement: S1 Fig — (DOCX) [file pone.0293837.s002.docx]

Appendix A – Alternative model

*Figure S2*


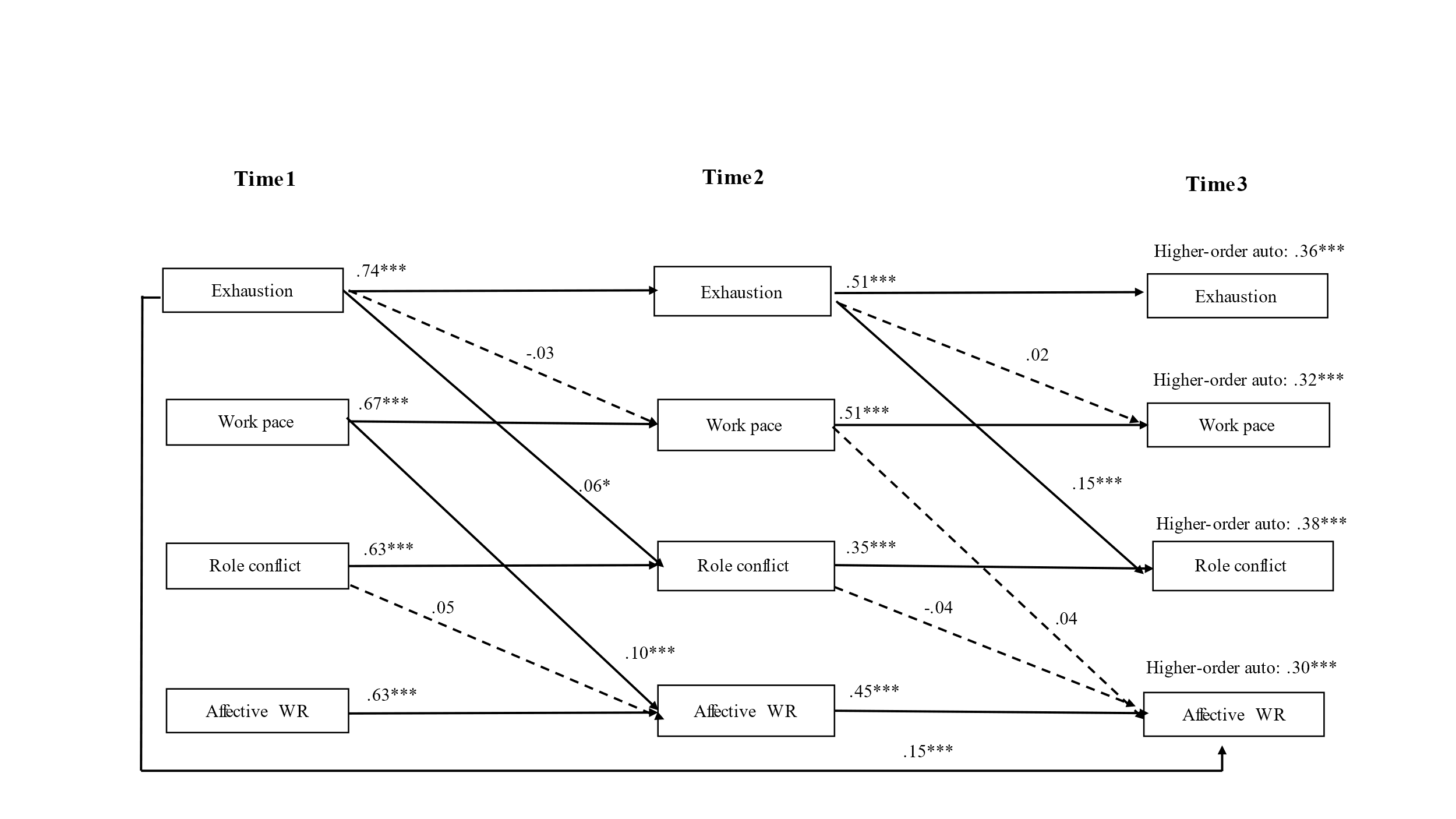


*Note.* Cross-lagged standardized regression weights for alternative model. Solid arrows indicate significant paths and dashed arrows indicated non-significant paths. Affective WR = Affective work rumination. Higher-order auto= Higher-order autoregressive effects between Time 1 and Time 3. All exogenous variables were allowed to correlate, and all residuals of the endogenous variables were allowed to correlate within each time point, for clarity reasons these effects are not depicted in the figure.

* *p*<.05, ***p*<.01, ****p*<.001.
